# Thermodynamic instability of viral proteins is a pathogen-associated molecular pattern targeted by human defensins

**DOI:** 10.1038/srep32499

**Published:** 2016-09-01

**Authors:** Elena Kudryashova, Pratibha C. Koneru, Mamuka Kvaratskhelia, Adam A. Strömstedt, Wuyuan Lu, Dmitri S. Kudryashov

**Affiliations:** 1 Department of Chemistry and Biochemistry, The Ohio State University, Columbus, OH 43210, USA; 2 Center for Retroviral Research, The Ohio State University, Columbus, OH 43210, USA; 3 College of Pharmacy, The Ohio State University, Columbus, OH 43210, USA; 4 Division of Pharmacognosy, Department of Medicinal Chemistry, Uppsala University, Biomedical Centre, Box 574, 75123 Uppsala, Sweden; 5 Institute of Human Virology and Department of Biochemistry and Molecular Biology, University of Maryland School of Medicine, Baltimore, MD 21201, USA; 6 Public Health Preparedness for Infectious Diseases Program, The Ohio State University, Columbus, OH 43210, USA

## Abstract

Human defensins are innate immune defense peptides with a remarkably broad repertoire of anti-pathogen activities. In addition to modulating immune response, inflammation, and angiogenesis, disintegrating bacterial membranes, and inactivating bacterial toxins, defensins are known to intercept various viruses at different stages of their life cycles, while remaining relatively benign towards human cells and proteins. Recently we have found that human defensins inactivate proteinaceous bacterial toxins by taking advantage of their low thermodynamic stability and acting as natural “anti-chaperones”, i.e. destabilizing the native conformation of the toxins. In the present study we tested various proteins produced by several viruses (HIV-1, PFV, and TEV) and found them to be susceptible to destabilizing effects of human α-defensins HNP-1 and HD-5 and the synthetic θ-defensin RC-101, but not β-defensins hBD-1 and hBD-2 or structurally related plant-derived peptides. Defensin-induced unfolding promoted exposure of hydrophobic groups otherwise confined to the core of the viral proteins. This resulted in precipitation, an enhanced susceptibility to proteolytic cleavage, and a loss of viral protein activities. We propose, that defensins recognize and target a common and essential physico-chemical property shared by many bacterial toxins and viral proteins – the intrinsically low thermodynamic protein stability.

Antimicrobial peptides (AMPs) in general and defensins in particular are major effectors of the innate immunity with a broad range of immune modulatory and direct antimicrobial activities^1^. Defensins are a family of short cationic amphiphilic, cysteine-rich AMPs found in vertebrates, invertebrates, and plants. Based on structural differences and tissue distribution these peptides are divided into three major classes α-, β- and θ-defensins. At the protein level, six α- and eleven β-defensins have been identified in humans^2^. θ-defensins, cyclic peptides found in Old World primates, are not produced in humans due to a premature stop codon in the mRNA transcript of the human θ-defensin pseudogene^3^. Humanized θ-defensins, retrocyclins (RCs), can be synthesized *in vitro* based on the sequence encoded by the human θ-defensin pseudogenes^4^.

Besides playing immunomodulatory roles^5^, human defensins exert direct antimicrobial activity by disorganizing bacterial cell membranes^6^, inhibiting the bacterial cell wall synthesis machinery^7^, and forming trapping nanonets around bacteria^8^. Importantly, defensins are the only recognized fast-response molecules that can neutralize a broad range of proteinaceous bacterial toxins, many of which are among the deadliest compounds on the planet and could harm or kill the affected organism if not immediately addressed. Thus, human defensins efficiently inhibit secreted toxins produced by over 30 pathogenic species, including enzymatic toxins and members of the largest family of pore-forming toxins, the cholesterol-dependent cytolysins^1^,^9^. Smaller in size, θ-defensins (including synthetic RCs) nevertheless share antibacterial and antitoxin activities with other natural defense peptides^10^.

For a decade, the question of how a small and structurally conserved group of peptides can neutralize a heterogeneous group of toxins with little to no sequential and structural similarities remained unresolved. Recently we found that the binding of toxins by human defensins and humanized RC peptides leads to local unfolding of the former and destabilization of their secondary and tertiary structures; this, in turn, increases toxins’ susceptibility to proteolysis and induces their precipitation^11^,^12^,^13^. We postulated that defensins recognize and target structural plasticity/thermodynamic instability, i.e. fundamental physico-chemical properties that unite many bacterial toxins and separate them from the majority of host proteins.

Intriguingly, there is a striking similarity between critical defensins’ determinates governing their antitoxin activities and those necessary for defensins’ binding to and neutralizing viral proteins: hydrophobicity, cationicity, and ability to dimerize/oligomerize^14^,^15^,^16^,^17^,^18^,^19^,^20^,^21^,^22^,^23^. Furthermore, many viral proteins display loosely packed cores (a hallmark of thermodynamic instability) that provide evolutionary advantage by conferring high interactive promiscuity and high mutational adaptability^24^,^25^. Accordingly, more than a dozen of various viruses are currently recognized as targets of defensins^26^. Moreover, human defensins are known to neutralize various enveloped and non-enveloped human viruses enigmatically acting at multiple different stages of viral invasion and replication^26^,^27^,^28^. While, some of the defensins’ effects can be explained by their lectin-like carbohydrate binding properties^29^, perturbation of lipid bilayers^30^ and/or modulation of host cell pathways^31^, we speculate that, in part, such multifaceted antiviral activity can be directly linked to the ability of defensins to promote unfolding of thermodynamically unstable pathogen-derived proteins. Thermodynamic instability as a recognition pattern of defensins may also help to explain immense amount of defensin-interacting protein targets both of viral and bacterial origins.

In the present study, we employed biochemical/biophysical approaches and functional assays to examine the defensins’ effects on a variety of viral proteins. We found that α- and θ-defensins potentiate unfolding of several viral proteins leading to their precipitation and loss of activity.

## Results

### In the presence of defensins several viral proteins demonstrate lower thermal stability

**Figure 1:**
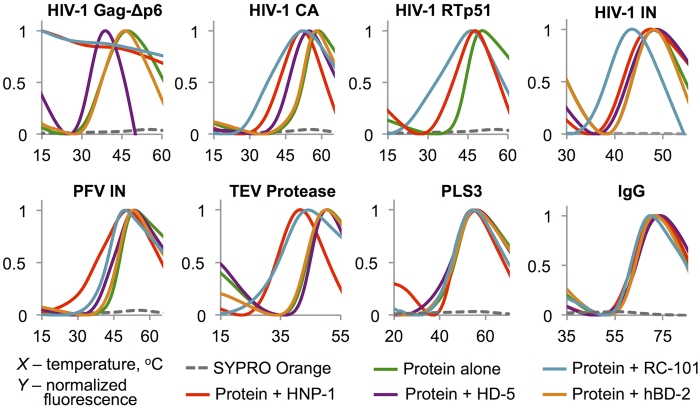
α- and θ-Defensins facilitate unfolding of viral proteins in DSF experiments. Thermal denaturation profiles of HIV-1 proteins Gag-Δp6 (10 μM), CA (20 μM), RTp51 (10 μM), and IN (5 μM), as well as PFV IN (7 μM) and TEV protease (5 μM) were shifted toward lower temperatures in the presence of 3-fold molar excess of HNP-1 or 5-fold molar excess of RC-101 over the viral proteins. HD-5 caused destabilization of HIV-1 proteins Gag-Δp6 and CA and PFV IN. β-Defensin hBD-2 had no effect on any of the tested proteins. Human structural protein plastin-3 (PLS3) and serum IgG were not significantly affected by the defensins. See also Table 1.

To test the hypothesis that defensins can promote unfolding of viral proteins we employed differential scanning fluorimetry (DSF) using the environmentally sensitive dye SYPRO Orange characterized by a dramatically enhanced quantum yield in hydrophobic environments (e.g. hydrophobic interior of a melted protein)^32^. We analyzed the effects of defensins on thermal melting profiles of several viral proteins from human immunodeficiency virus type 1 (HIV-1), prototype foamy virus (PFV) and tobacco etch virus (TEV). All tested proteins fall into two groups: those, whose melting was notably potentiated by defensins, and all others, whose melting profiles could not be accurately assessed due to an initially high degree of instability. The latter group was composed of several HIV-1 proteins (viral infectivity factor Vif, negative regulatory factor Nef, matrix protein MA, and protease), for all of which the SYPRO Orange fluorescence was heightened at the lowest tested temperatures (data not shown), which is characteristic for proteins in a molten globule state^33^, or with high surface hydrophobicity index (e.g. bovine serum albumin^11^). The former group of viral proteins, clearly affected by defensins, included HIV-1 Gag-Δp6, HIV-1 capsid (CA), HIV-1 reverse transcriptase (RT p51), HIV-1 integrase (HIV-1 IN), PFV integrase (PFV IN), and TEV protease. The effects of the following defensins were assessed: human neutrophil α-defensin peptide HNP-1, human enteric α-defensin HD-5, human β-defensins hBD-1 and hBD-2, and humanized retrocyclin RC-101 (Fig. 1, Table 1, Supplementary Fig. S1). HNP-1 and RC-101 were consistently - more potent than other defensins, whereas the most susceptible of all viral proteins was Gag-Δp6 (Fig. 1). Destabilization of Gag-Δp6 by HNP-1 and RC-101 occurred at the lowest tested temperature. β-defensins hBD-1 (Supplementary Fig. S1) and hBD-2 (Fig. 1) did not potentiate protein unfolding to any noticeable extent at the standard assay conditions (i.e. under physiological salt concentrations). Consistent with its localization in primarily hypotonic compartments (skin and oral mucosa), the bactericidal effects of hBD-2 are known to be attenuated by salt^34^. Therefore, we tested thermal denaturation of HIV-1 Gag-Δp6, HIV-1 CA, PFV IN, and TEV protease in the absence of salt and observed no significant changes in melting profiles of these proteins in the presence of either hBD-1 or hBD-2 (Supplementary Fig. S1). Given the only marginal bacterial toxin unfolding activity of hBD-2 in our previous investigation^11^, we can conclude that in contrast to α- and θ-defensins, β-defensins have overall low ability to potentiate unfolding of thermodynamically unstable proteins.

### Defensins increase susceptibility of viral proteins to limited proteolysis

**Figure 2:**
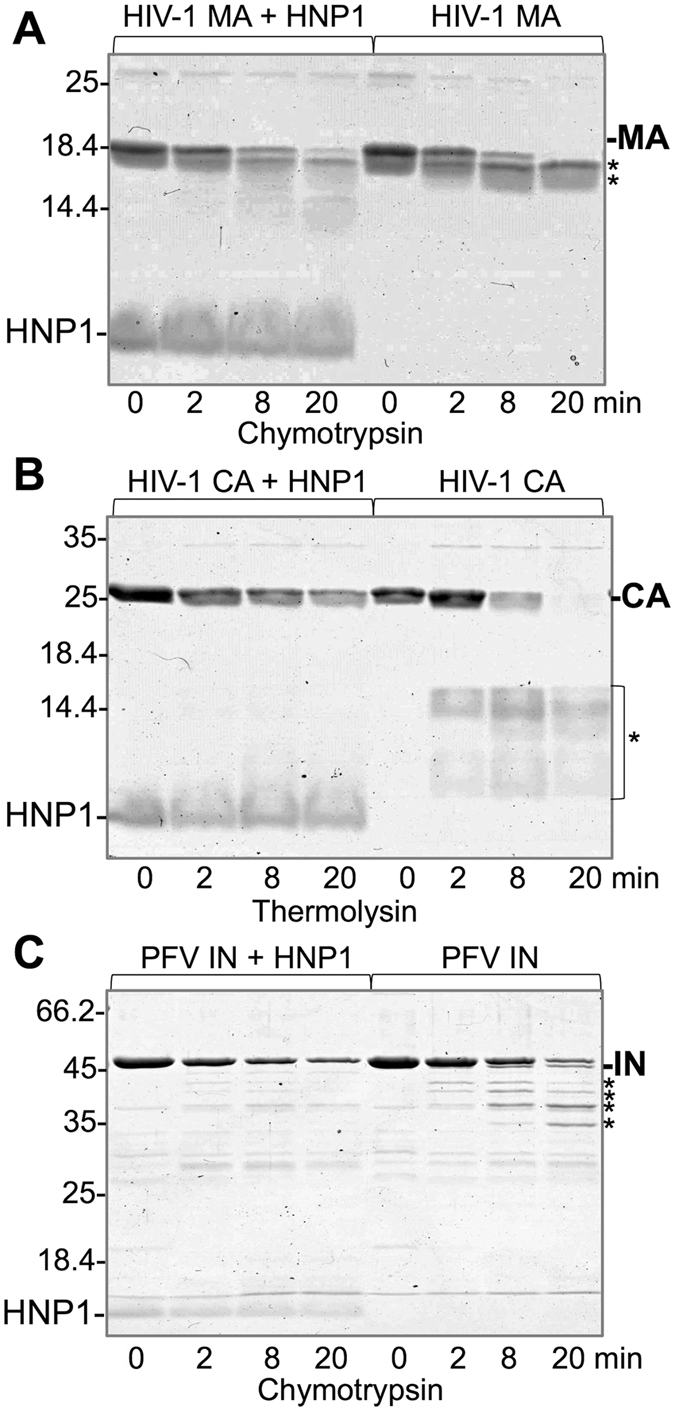
Limited proteolysis suggests exposure of additional cleavage sites on viral proteins in the presence of HNP-1. 10 μM HIV-1 MA (**A**), 10 μM HIV-1 CA (**B**) and 5 μM PFV IN (**C**) were subjected to proteolytic cleavage by low doses of chymotrypsin (1:100 w/w enzyme to substrate ratio) or thermolysin (1:200 w/w enzyme to substrate ratio) for the indicated periods of time. Asterisks indicate notable proteolytic products detected in the absence, but not presence of HNP-1. The full-length protein in each case, both in the presence and absence of HNP-1, gets degraded as seen by the decreasing amounts of the corresponding bands on the gels.

To further examine defensin-induced conformational changes of viral proteins, we probed their structural stabilities by limited proteolysis^35^ using chymotrypsin and thermolysin – proteolytic enzymes, which preferentially hydrolyze the peptide bonds at the C-end side of bulky hydrophobic residues. In the absence of HNP-1, MA and CA proteins from HIV-1 and IN from PFV subjected to limited proteolysis showed a specific pattern of well-defined proteolytic products (denoted by asterisks on Fig. 2) typical for cleavage at a few highly accessible unstructured sites. In contrast, addition of HNP-1 resulted in a drastic reduction in the detectable amounts of cleaved products, despite that the amount of the full-length protein was decreased over time due to proteolysis (Fig. 2). This result resonates with the ability of HNP-1 to promote unfolding of several bacterial toxins and thus increase a number of residues accessible for cleavage generating multiple proteolytic products of various sizes scattered throughout a lane on a gel^11^. In one case, this effect is evident as a smear of HIV-1 MA protein in the presence of HNP-1 instead of the well-defined bands evident in its absence (Fig. 2A).

### Defensins promote precipitation of viral proteins

**Figure 3:**
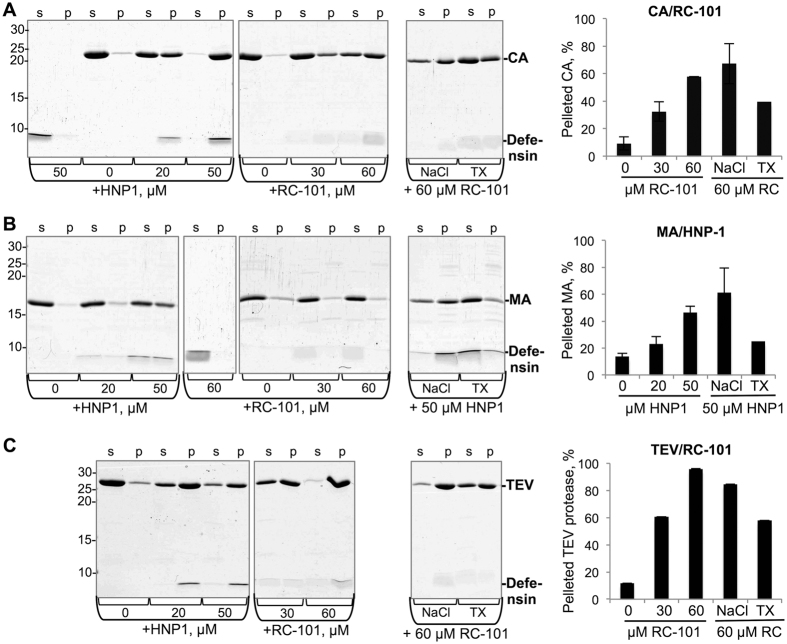
Defensin-induced precipitation of viral proteins. 10 μM proteins (HIV-1 CA (**A**), HIV-1 MA (**B**), and TEV protease (**C**)) were incubated in the absence or presence of different concentrations of HNP-1 or RC-101 and subjected to centrifugation. In additional experiments, to test the solubility of the defensin-induced precipitates, either 0.5 M NaCl (indicated as “NaCl”) or 0.5% of Triton X-100 (indicated as “TX”) was added to the samples following the incubation of the proteins with the highest concentration of defensins and before the centrifugation. Supernatant (*s*) and pellet (*p*) fractions were resolved on SDS-PAGE. Graphs show the quantitation of the amount of proteins in the pellet fractions expressed in percent of total protein content.

Due to the unfolding of viral proteins caused by defensins, hydrophobic interior of the affected proteins becomes exposed to solution, which in turn may lead to protein aggregation. Therefore, we conducted precipitation assays, where HIV-1 MA and CA proteins and TEV protease were incubated in the presence or absence of defensins and subjected to high-speed centrifugation to sediment protein aggregates. HNP-1 caused dose-dependent precipitation of all tested viral proteins (Fig. 3), indicative of the promoted exposure of the hydrophobic interior, in consistence with our limited proteolysis results. RC-101 caused aggregation of CA and TEV protease, but not MA, which remained mainly in solution. Addition of sodium chloride (0.5 M final concentration) to the precipitated viral proteins did not reverse the defensin-induced aggregation of these proteins, while the addition of non-ionic detergent Triton X-100 (0.5% final concentration) partially reversed the precipitation (Fig. 3). This is indicative of the prevalence of hydrophobic rather than electrostatic interactions in these aggregates.

### In the presence of RC-101, tryptophan residues of viral proteins are more exposed to collisional quenchers

**Figure 4:**
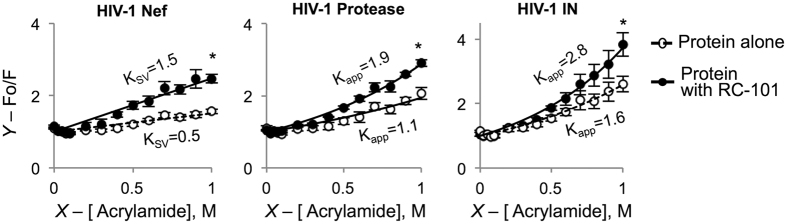
Collisional quenching of Trp fluorescence by acrylamide reveals greater accessibility of viral proteins to the quencher in the presence of RC-101. Stern-Volmer plots for HIV-1 proteins (2 μM) - Nef, protease, and IN – in the absence (open circles) and in the presence of 5-fold molar excess of RC-101 (solid circles). *X*-axes are molar concentrations of the quencher (acrylamide), *Y*-axes – ratio of the Trp fluorescence in the absence of the quencher (F_0_) and in its presence (F). Dynamic Stern-Volmer coefficients (K_SV_) are provided for Nef protein. In contrast, quenching of protease and IN proteins show both strong dynamic and static components reflected in a characteristic upward curvature of Stern-Volmer plots. For these proteins, apparent Stern-Volmer coefficients (K_app_) at acrylamide concentration equal 1 M are shown. Increased K_SV_ (or K_app_) in the presence of RC-101 imply higher accessibility of Trp residue(s) to the quencher. Asterisk indicates statistically significant difference (*P*<0.05).

Collisional quenching of tryptophan (Trp) fluorescence is an effective method for monitoring conformational changes in the protein structure^36^. Trp fluorescence of HIV-1 proteins (protease, Nef and IN) in the presence and absence of RC-101 was assessed and plotted as a function of increasing concentrations of a quencher (acrylamide) (Fig. 4). The linearity of the Stern-Volmer plots for HIV-1 Nef protein allowed for determining Stern-Volmer constant (K_SV_), which was significantly higher in the presence of RC-101 suggesting greater accessibility of the protein’s interior for the quencher (Fig. 4). For HIV-1 protease and IN the Stern-Volmer plots demonstrated an upward curvatures, characteristic for the presence of both dynamic and static components of the quenching^37^. Therefore, the apparent quenching coefficients (K_app_) were calculated at 1 M quencher concentration. In both cases (for HIV-1 protease and IN) K_app_ was significantly higher in the presence of RC-101 (Fig. 4), in agreement with the defensin’s ability to perturb integrity of viral proteins.

### Defensins inhibit functional activity of TEV protease

**Figure 5:**
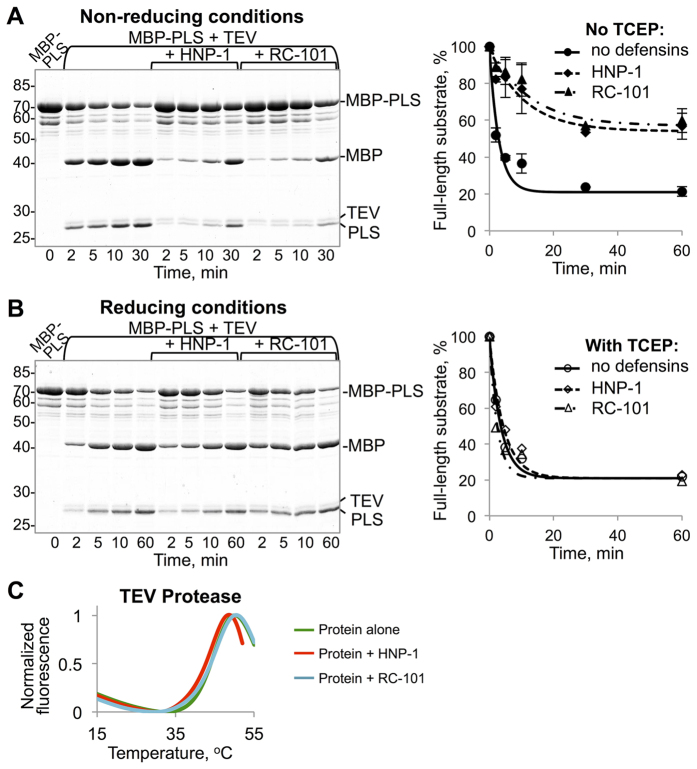
Defensins’ disulfide bonds are essential for the inhibition of TEV protease activity. (**A**) Activity of TEV protease was assessed in the absence of defensins and following the incubation of TEV protease with HNP-1 (3-fold molar excess) or RC-101 (5-fold molar excess). Cleavage of MBP-PLS (5 μM; 20:1 w/w ratio to TEV protease) was monitored by SDS-PAGE as a reduction of the full-length substrate protein MBP-PLS expressed in percent and plotted versus time. (**B**,**C**) Under reducing conditions (10 mM TCEP), defensins do not affect the activity (**B**) or melting profile (**C**) of TEV protease.

The defensin-induced unfolding and precipitation interferes with the activity of TEV protease, which was assessed using an artificial substrate comprised of maltose binding protein (MBP) and actin-binding domain of PLS3 fused together through a linker containing the TEV protease cleavage site (Fig. 5A). Following HNP-1 or RC-101 treatment, TEV protease activity was decreased ~3–4 times, compared to the untreated protease, as calculated based on the decline of the activity rate measured at the two-minute time point.

Three characteristic disulfide bonds stabilize the defensins’ structure and are essential for some, but not all functions of the peptides. Thus, unstructured HNP-1 and HD-5 analogs lacking cysteines show preserved antibacterial activity against Gram-negative bacteria, but are completely inactive against Gram-positive bacteria^9^. This is likely because the preserved defensin structure is required in the later case, when the cell wall synthesis machinery is targeted, but not in the former case, when defensins directly affect membranes of Gram-negative bacteria. In contrast, reduction of disulfide bonds activates hBD-1 antimicrobial activity against both Gram-negative and Gram-positive bacteria^38^. Disulfide bonds in hBD-3 are required for its chemotactic activity (i.e. binding and activation of the chemokine receptor CCR6), but dispensable for its antimicrobial activity^39^. Furthermore, the ability of an epithelial α-defensin HD-5 to inactivate adenovirus was not reproduced by a cysteine-deficient HD-5 analog^40^. Accordingly, we found that reduction of HNP-1 and RC-101 disulfide bonds with TCEP resulted in complete loss of the defensins’ ability to inhibit the proteolytic activity of TEV protease (Fig. 5B) and to promote its unfolding (Fig. 5C). This is in agreement with our previous finding that native oxidized state of RC-101 is essential for its antitoxin activity^12^. Similarly, reduced β-defensins hBD-1 and hBD-2 had no effect on thermal melting of TEV protease and HIV-1 Gag-Δp6 and CA (Supplementary Fig. S1), suggesting that the enhanced antibacterial activity of reduced hBD-1^38^ arises from different mechanisms.

### Defensins interfere with the integration activity of HIV-1 integrase

**Figure 6:**
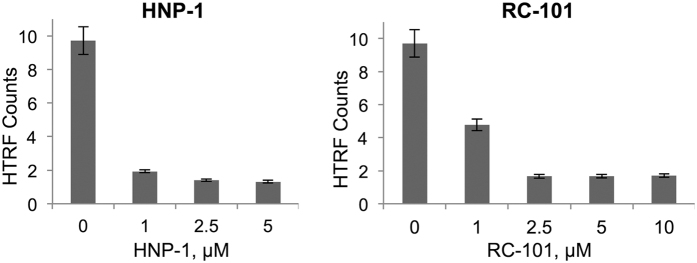
HIV-1 integrase (IN) activity is inhibited by HNP-1 and RC-101. Strand transfer activity of HIV-1 IN (400 nM) in the absence and presence of various defensin concentrations (1–10 μM) was determined by homogenous time-resolved fluorescence (HTRF) technique.

We tested whether the defensin-induced unfolding of HIV-1 IN, evidenced from DSF (Fig. 1) and collisional quenching (Fig. 4) experiments, interferes with the integration activity of this protein. HIV-1 IN catalyzes concerted integration of the viral DNA ends into human chromosome, which defines a point of “no return” in establishing HIV-1 infection. Therefore, we conducted a homogeneous time-resolved fluorescence (HTRF)-based assay to probe a strand transfer activity of HIV-1 IN^41^,^42^ in the absence and in the presence of HNP-1 and RC-101. The assay measures fluorescence resonance energy transfer between target DNA and viral donor DNA upon formation of the strand transfer product. Figure 6 shows that both HNP-1 and RC-101 inhibited the strand transfer activity of IN.

### Cyclotides do not promote unfolding of viral proteins

**Figure 7:**
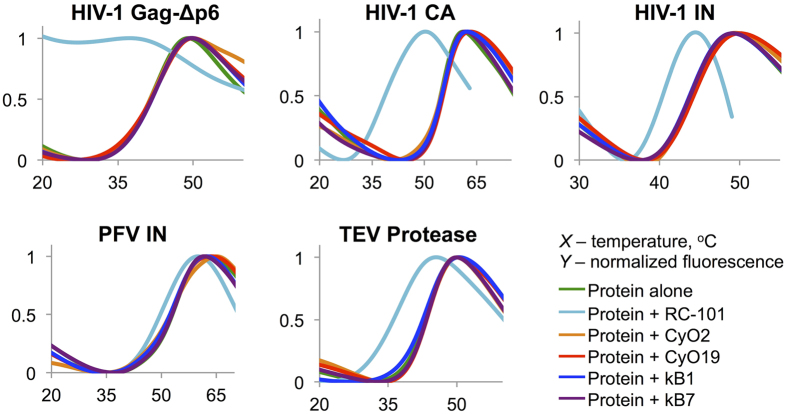
Cyclotides do not potentiate unfolding of the tested viral proteins. DSF thermal denaturation profiles of viral proteins (HIV-1 Gag-Δp6, HIV-1 CA, HIV-1 IN, PFV IN, and TEV protease) were collected in the absence or in the presence of 5-fold molar excess of defense peptides (θ-defensin RC-101 and cyclotides cyO2, cyO19, kB1, kB7).

Cyclotides are plant-derived antimicrobial peptides that share structural similarities with defensins. Both have backbones of a similar length with a tightly packed tertiary structure. Like the θ-defensins, cyclotides have a head-to-tail cyclic backbone interlocked by three disulfide bonds, although arranged in a “cyclic cystine knot” motif, as opposed to a “cyclic ladder” arrangement of θ-defensins^10^. Similar to defensins, cyclotides have a broad range of antimicrobial activities^43^. Both peptide families interact with negatively charged phospholipid membranes (e.g. bacterial cell membranes) via amphiphilic patches leading to membrane permeabilization by related mechanisms^44^,^45^. Furthermore, both, defensins^26^,^46^ and cyclotides (including kalata B1 used in this study)^47^, exhibit antiviral activity by largely unknown mechanisms. However, in contrast to defensins, none of the four cyclotides used in this study (cyO2, cyO19, kB1, and kB7) demonstrated an ability to potentiate unfolding of viral proteins (HIV-1 Gag-Δp6, HIV-1 CA, HIV-1 IN, PFV IN, and TEV protease; Fig. 7) or a thermolabile bacterial toxin (actin crosslinking domain – ACD – from *Vibrio cholerae*; Supplementary Fig. S2). Therefore, the ability to promote unfolding of thermodynamically unstable viral proteins (present study) and bacterial toxins^11^,^12^ thus far is restricted to α- and θ-defensins. On the other hand, β-defensins showed only marginal activity against bacterial toxins^11^, whereas cyclotides were inactive against all examined viral and bacterial proteins.

## Discussion

High conformational freedom confers critical functional advantages to many bacterial proteinaceous toxins: with only a minimal input of free energy, high protein plasticity allows dramatic conformational changes essential for transition from soluble to a membrane-integrated form (pore-forming toxins), or a temporarily unfolded form (pore-crossing toxins). Yet, conformational plasticity comes with a price of thermodynamic instability, which can be semi-selectively targeted for elimination by host immune effector molecules – defensins. Remarkably, the list of marginally stable pathogenic proteins extends well beyond bacterial toxins and encompasses many viral proteins. While for the majority of host proteins the equilibrium between folded and unfolded states is strongly shifted towards the former at the physiological range of temperatures, this is not the case for marginally stable pathogenic proteins, whose transition point from folded to melted states is typically only few degrees above the body temperature of their hosts^48^. Our previous findings suggested that defensins are capable of promoting partial unfolding of marginally stable bacterial toxins^11^. The present study demonstrates that this ability of defensins extends towards viral proteins. We hypothesize that the common property of bacterial toxins and viral proteins targeted by defensins is their marginal thermodynamic stability essential for conformational plasticity. While in case of bacterial toxins, this property is tentatively dictated by the necessity to undergo dramatic conformational transitions with minimal input of energy, thermodynamic instability of viral proteins provided additional benefits. Indeed, for many viral proteins low thermodynamic stability is the way of maintaining high interaction potential and high mutational adaptability^24^,^25^ – essential properties dictated by small-size genomes and high evolutionary pressure of host immune systems, respectively. Accordingly, various viral proteins have been reported to exist at physiological temperatures in a molten globule states, e.g. HIV-1 transactivating nuclear protein Rev^49^, herpes simplex virus (HSV) triplex protein VP23^50^, influenza virus protein NS2^51^, potato virus A genome-linked protein VPg^52^.

We reported here that Gag, capsid, integrase, reverse transcriptase, Nef, and matrix proteins of HIV-1 as well as integrase of PFV and protease of TEV are all destabilized/unfolded by human defensins as demonstrated by at least one of the following techniques: DSF, intrinsic Trp accessibility to solution quenchers, and/or limited proteolysis. Due to the limitations for individual methods, not every protein could be tested by all of the approaches. For example, the number and location of Trp residues in a protein can hinder the assessment of its conformational changes by collisional quenching. Thus, changes in HIV-1 MA and CA proteins were revealed by limited proteolysis and precipitation analysis for both proteins and by DSF for CA, but not by collisional quenching. Likewise, DSF cannot be applied to proteins that actively bind an environmentally sensitive dye in their ground state. The later can be either due to inherently high surface hydrophobicity (like in serum albumin) or the result of dye penetration into a loosely packed hydrophobic core even at low temperatures (like with proteins existing in a molten globule state). Thus, unattainable by DSF changes in some proteins (e.g. Nef and protease of HIV-1) were observed for these proteins in the collisional quenching experiments (Fig. 4). Even though the defensin-susceptible viral proteins tested in the present study are located underneath the viral membrane envelope, membrane-disorganizing and pore-forming abilities of defensins make all of them potentially accessible for the attack. Alternatively, these proteins might be targeted intracellularly, as defensins have been demonstrated to retain some of their antiviral activity even after the viral entry^28^.

The tenet that defensins promote unfolding of viral proteins, as was observed for bacterial toxins, greatly contributes to multifaceted, direct and indirect antiviral mechanisms of defensins and provides appealing logical explanation to hitherto enigmatic ability of HNP-1 to inhibit HIV-1 and HSV infections at multiple steps^27^,^28^. In fact, many previous observations are in excellent agreement with the proposed ability of defensins to promote unfolding of viral proteins. Thus, the neutrophil α-defensin HNP-1 has been shown to prolong refolding lifetime of gp41 intermediates in pre-hairpin conformation, as demonstrated by defensin-improved access of neutralizing antibodies and peptides to otherwise hidden epitopes^53^. Because protein unfolding is accompanied by exposure of hydrophobic surfaces, normally buried in the protein’s interior, it is likely to be accountable for defensin-induced aggregation of many pathogen-derived proteins, including gp41 peptides. Thus, HNP-1 and retrocyclin-1 (RC-1) impaired refolding of gp41 into the 6-helix bundle structure triggering their aggregation^27^. Moreover, circular dichroism analysis revealed that in the presence of RC-1 a characteristic α-helical spectrum of the gp41-derived peptide mixture of N36/C34 transformed into a random coil-like spectrum^54^, which can be interpreted as secondary structure destabilization by this peptide. RC-1 also inhibits dengue virus DENV-2 replication by interfering with the activity of its serine protease and this ability is greatly increased with elevated temperature^55^, i.e. under conditions enriching the fraction of partially unfolded protein intermediates that are selected and trapped by defensins. Similarly, we found that inhibition of TEV protease activity by both HNP-1 and RC-101 (Fig. 5A) resulted from the enzyme unfolding (Fig. 1) and precipitation by the defensins (Fig. 3C).

It is likely that the potent destabilizing effects imposed by defensins are pertinent at numerous physiological occasions and particularly upon dynamic structural transitions in the course of capsid assembly^50^,^56^, uncoating^57^, and membrane fusion^58^. Yet, we are far from proposing that the destabilization of purified viral proteins by defensins observed *in vitro* accurately represents the complexity of physiological conditions including, but not limited to, interactions of viral proteins with each other, viral nucleic acids, and host proteins. Thus, in apparent contradiction with our results, stabilization of viral capsids and prevention of their uncoating by defensins has been reported in several studies^59^,^60^,^61^. It is plausible however that this increased capsid stability may paradoxically result from defensin-induced structure perturbation of individual marginally stable capsid proteins, or even individual protein domains, leading to their respective inability to respond to environmental cues and go through proper conformational rearrangements. The exact mechanisms of protein function disruption by defensins are likely to differ in each particular case. For example, defensins may induce local unfolding and exposure of hydrophobic patches in the affected proteins leading to their unnatural interaction with each other and with hydrophobic patches of defensins. Such interactions would be mimicking precipitation but occurring locally, whereas the entire capsid structure can be enforced by these newly formed hydrophobic interactions. Alternatively, defensins may prevent proper conformational transitions in key capsid proteins. In line with these hypotheses, overall stabilization of Ad5.F35 capsid by HD-5 defensin has been accompanied by signs of local disorganization and greater conformational flexibility of individual capsid proteins (hexon, penton base, and fiber) in CryoEM studies^40^,^59^.

Of the four tested groups of antimicrobial peptides (Supplementary Table S1), only α- and θ-defensins demonstrated the strong ability to promote unfolding of viral proteins and bacterial toxins, whereas β-defensins (hBD-1 and -2) and plant cyclotides (cyO2, cyO19, kB1, and kB7) failed to reproduce these effects. This is intriguing given that the majority of the peptides share with α-defensins many molecular properties shown to contribute to inactivation of bacterial effectors: three disulfide bonds, cationicity, amphiphilicity, and ability to assemble into dimers/oligomers^14^,^15^,^16^,^17^,^18^,^19^,^20^,^21^,^22^,^23^,^43^. However, because the mechanism of protein destabilization by defensins is not known in detail, the precise roles of each of these features are obscure and a proper, activity-enabling balance between them cannot be currently predicted. Thus, it has been demonstrated that a conserved Trp-26 residue is essential for HNP-1 activity as a contributor to hydrophobicity for interaction with target molecules and for formation of dimers^15^. However, retrocyclins, which are similarly active against viral (current study) and bacterial effectors^12^, do not have tryptophans, suggesting that the role of this residues is fulfilled by other structural elements. In the absence of a detailed and quantifiable model, we can provide only speculative explanation to the observed differences in activities of the peptides. First, all basic residues in HNP-1 and HD-5 “active” defensins are represented by arginines, whereas those of hBD-1 and hBD-2 are represented mostly by lysines and some histidines. Since three nitrogen atoms of the arginine guanidinium group enable more freedom in establishing electrostatic interactions and hydrogen bonds as compared to lysines^62^,^63^, this difference might be essential in a more potent docking of the peptides to hydrophilic elements of the effector proteins. Second, the tested β-defensins are somewhat bulkier than α-defensins (36–41 a.a. versus 30–32 a.a., respectively; Supplementary Table S1), which may make them less capable of inserting into protein pockets and interfering with proper folding. From this perspective, high destabilizing activity of θ-defensins correlates with their overall smaller size (18 a.a). Other factors to be considered are densities of basic and hydrophobic residues (Supplementary Table S1). Among all the tested peptides, RC-101 has the highest density of basic residues (2.22 per 10 residues), 3 out of 4 of which are arginines, while their density in cyclotides is the lowest and varies from 0.34 to 1.0 per 10 residues. Hydrophobicity index seems to correlate with the ability to destabilize viral proteins in case of RC-101 and HNP-1 (high hydrophobicity index and high potency) and β-defensins (low hydrophobicity and low potency), while in direct comparison it fails to correlate with those of cyclotides (high hydrophobicity and low potency) and HD-5 (low hydrophobicity and high potency). Finally, the ability to form dimers/higher order oligomers and the affinity of the subunits within the dimer/oligomer to each other is yet another parameter that is likely to influence the ability of the immune peptides to promote unfolding of marginally stable effector proteins. However, these properties of immune peptides are poorly investigated and will require careful characterization before more definite conclusions can be made. A fine balance between all the above properties of the immune peptides appears to be essential for rendering them active and as such should be addressed in future experimental and computational studies.

In conclusion, we propose that conformational plasticity is the key feature that unites various bacterial toxins and viral proteins. As in a classical scheme when the greatest strength is the other side of the utmost weakness, this property is crucial for pathogenicity of these proteins, but it also renders them susceptible to destabilizing effects of human innate peptides.

## Methods

### Antimicrobial peptides

Defensins HNP-1, HD-5, hBD-1, hBD-2, and humanized retrocyclin RC-101 were prepared by solid-phase peptide synthesis and the correct folding was ensured as described previously^39^,^64^,^65^,^66^. The cyclotides cyO2 and cyO19 were isolated from a *Viola odorata* L. extract, while the cyclotides kB1 and kB7 were isolated from an *Oldenlandia affinis* D.C. extract according to the methods previously described^45^,^67^. The peptides were of >95% purity as assessed by mass spectrometry and reversed-phase HPLC.

### Pathogen-derived proteins

HIV-1 matrix (HIV-1 MA) protein and Gag-Δp6 were purified as described^68^,^69^. HIV-1 capsid (HIV-1 CA), HIV-1 integrase (HIV-1 IN) and PFV integrase (PFV IN) were purified as described^70^,^71^,^72^. Proteolytic domain of 6-His-tagged TEV protease was expressed and purified according to a standard procedure using Talon cobalt resin (Clontech). The following reagents were obtained through the NIH AIDS Research and Reference Reagent Program, Division of AIDS, NIAID, NIH: HIV-1_HXB2_ p51 reverse transcriptase recombinant protein (cat. #2896; from Dr. Stuart Le Grice^73^), HIV-1 protease (cat. #11781), HIV-1 Nef recombinant protein (cat. #11478), and recombinant HIV-1 Vif (Baculovirus) (cat. #11050). Actin crosslinking domain (ACD) from *Vibrio cholerae* MARTX toxin was purified as described previously^74^.

### Human proteins

Plastin-3 (PLS3) was purified as described^75^. Purified human IgG was obtained from Sigma (cat. #I4506). MBP-PLS, an actin-binding domain of PLS3 in-frame with maltose binding protein (MBP) and TEV protease cleavage site, was purified using amylose resin (New England Biolabs) according to the manufacturer instructions.

### Differential scanning fluorimetry (DSF)

Viral protein samples (5–20 μM) in a 20 mM 4-(2-hydroxyethyl)-1-piperazineethanesulfonic acid (HEPES) buffer (pH 7.5) supplemented with 150 mM NaCl and 1x SYPRO Orange dye (Invitrogen) were subjected to temperature denaturation in the absence or presence of defensins. HNP-1, HD-5, hBD-1 and hBD-2 were used at a 3-fold excess to the viral proteins; RC-101 was used at a 5-fold excess. A higher molar ratio of RC-101 was used to compensate for its smaller size (18 a.a. RC-101 versus 30–42 a.a. α- and β-defensins). Temperature melting profiles were acquired with a CFX96 Touch Real-Time PCR Detection System (Bio-Rad). Melting temperatures for proteins alone and in the presence of different defensins were calculated as described previously^76^.

### Limited proteolysis

Limited proteolysis was conducted as described previously^11^. Briefly, viral proteins (5–10 μM) were mixed with 3-fold molar excess of HNP-1 (i.e. 15–30 μM) in a 20 mM HEPES buffer (pH 7.5) supplemented with 100 mM NaCl and 5 mM CaCl_2_. Cleavage was achieved by the use of chymotrypsin (1:100 w/w ratio to protein) or thermolysin (1:200 w/w ratio) at 37 °C for varying amount of time. The reactions were stopped by adding reducing sample buffer (50 mM Tris, pH 6.8, 10% glycerol, 2% sodium dodecyl sulfate (SDS), 100 mM β-mercaptoethanol, 0.1% bromophenol blue) supplemented with 2 mM phenylmethylsulfonyl fluoride (PMSF) and 10 mM ethylenediaminetetraacetic acid (EDTA) and boiling for 5 min. The protein samples were resolved on SDS-PAGE.

### Precipitation assay

Precipitation of HIV-1 MA, HIV-1 CA, and TEV protease was assessed by differential ultracentrifugation. Prior to centrifugation, 10 μM of each protein was incubated in the absence or presence of 20 and 50 μM of HNP-1 or 30 and 60 μM of RC-101 for 30 min at 37 °C in a 20 mM HEPES buffer (pH 7.5) with 100 mM NaCl. In additional experiments, either 0.5 M NaCl or 0.5% of Triton X-100 was added following the incubation of the proteins with the defensins, to test the solubility of the defensin-induced aggregates. All samples were centrifuged using TLA-100 rotor in Optima TL-100 ultracentrifuge (Beckman Coulter) at 280,000 g for 30 min at 4 °C. The supernatant (soluble) and pellet (precipitated) fractions were collected, supplemented with 1/3^rd^ of the volume of 4x reducing sample buffer (200 mM Tris, pH 6.8, 40% glycerol, 8% SDS, 400 mM β-mercaptoethanol, 0.4% bromophenol blue), pellets resuspended, and samples resolved on SDS-PAGE.

### Collisional quenching of tryptophan fluorescence

Trp fluorescence was measured using a multifunctional plate reader Infinite M1000Pro (Tecan) with excitation and emission wavelengths 295 and 328 nm, respectively. Viral proteins were diluted to 2 μM in 20 mM HEPES (pH 7.5), 150 mM NaCl with or without addition of 5-fold molar excess of RC-101 (i.e. 10 μM) and titrated with increasing amounts of freshly prepared acrylamide solution in the same buffer. Data are presented as Stern-Volmer plots, where the ratios of fluorescence intensities (F_0_/F) in the absence (F_0_) and presence (F) of a given quencher (acrylamide) concentration were plotted against quencher concentration ([Q])^36^. Stern-Volmer constants (*K*_*SV*_) were calculated according to the Stern-Volmer equation:


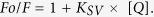


In some cases, to account for both strong dynamic and static components of quenching, which resulted in a characteristic upward curvature of Stern-Volmer plots, the modified Stern-Volmer equation was used:


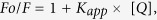


where *K*_*app*_ is an apparent quenching constant encompassing both dynamic and static constants^37^.

### TEV protease activity assay

TEV protease was incubated in the presence or absence of HNP-1 (in a 3-fold molar excess to TEV protease) or RC-101 (in a 5-fold molar excess to the protein) at 37 °C for 30 min. Next, 5 μM of an artificial substrate protein MBP-PLS (containing maltose binding protein and actin-binding domain of PLS3 connected through a linker containing TEV protease cleavage site) in a 20 mM HEPES buffer (pH 7.5) with 150 mM NaCl was incubated with the pre-treated TEV protease (at 1:20 w/w ratio to MBP-PLS) at 37 °C for 2–60 min. The reactions were stopped by boiling in reducing sample buffer; proteolytic products were resolved on SDS-PAGE. For reducing condition experiments, the reaction buffer was supplemented with 10 mM tris(2-carboxyethyl)phosphine (TCEP) and the defensins were pre-incubated with 10 mM TCEP for one hour at room temperature before co-incubation with the TEV protease. Note, the ammonium salt of TCEP (Sigma #646547) used in this study has neutral pH (7.0). Care was taken to verify that the final pH of the working solutions was not changed after the addition of TCEP.

### Homogenous time-resolved fluorescence (HTRF)-based integration activity assay

To test the HIV-1 integrase (IN) activity in the presence and absence of defensins, previously developed HTRF technique^41^,^42^ was used with some modifications. HIV-1 IN (400 nM) was incubated with various concentrations of HNP-1 and RC-101 (1–10 μM) for 30 min at room temperature in the reaction buffer (20 mM HEPES (pH 7.5), 10 mM MgCl_2_, 10% glycerol, 0.05% Brij-35, 0.1 mg/mL BSA). Following the incubation with the defensins, a 5′-Cy5-labelled 21-mer viral donor DNA (200 nM) and 3′-biotinylated target DNA (20 nM) were added to the reaction. The strand transfer reaction was allowed to proceed for three hours at 37 °C and stopped by the addition of 2x development solution (20 mM EDTA, 0.05% Brij-35, 1 mg/mL BSA, 1 M NaCl, 4 nM europium chelate-streptavidin (Eu-SA)). 0.5 M NaCl (final concentration in the development solution) allows dissociating of all DNA from IN to prevent FRET signal from non-covalently associated donor and target DNA. Following an overnight incubation at 4 °C, the HTRF signal was recorded with an EnSpire multimode plate reader (PerkinElmer).

### Statistical analysis

Throughout, error bars represent standard errors of the mean values. Statistical significance was determined by two-tailed Student’s *t*-test: results were considered significant at *P*-values less than 0.05.

## Additional Information

**How to cite this article**: Kudryashova, E. *et al.* Thermodynamic instability of viral proteins is a pathogen-associated molecular pattern targeted by human defensins. *Sci. Rep.* **6**, 32499; doi: 10.1038/srep32499 (2016).

## Supplementary Material

Supplementary Information

## Figures and Tables

**Table 1:** Effects of defensins on melting temperatures (T_m_, °C) of viral and host proteins calculated from DSF.

	Protein alone	+ RC-101	+ HNP1	+ HD5	+ hBD2
HIV-1 Gag-Δp6	38.6 ± 0.3	n/a	n/a	34.3 ± 0.6	38.9 ± 0.2
HIV-1 CA	51.9 ± 0.1	41 ± 0.1	44.2 ± 0.5	47.6 ± 0.2	51.4 ± 0.2
HIV-1 RTp51	44.4 ± 0.1	37 ± 0.2	40.3 ± 0.1	n/d	n/d
HIV-1 IN	42.5 ± 0.5	38.3 ± 0.6	41.0 ± 0.3	42.0 ± 0.5	43.2 ± 0.9
PFV IN	47.6 ± 0.1	42.5 ± 0.1	43.3 ± 0.1	45.3 ± 0.1	46.5 ± 0.2
TEV Protease	43.7 ± 0.2	33.8 ± 1.8	34.9 ± 0.2	44.7 ± 0.2	44.0 ± 0.2
PLS3	47.8 ± 0.1	45.7 ± 0.1	46.8 ± 0.2	47.5 ± 0.1	47.4 ± 0.1
IgG	63.8 ± 0.2	63 ± 0.1	64.5 ± 0.1	65.2 ± 0.2	64 ± 0.2

n/a – Not applicable (DSF curve does not have a peak).

n/d – Not determined.
