# Supplementary material for: Thermodynamic instability of viral proteins is a pathogen-associated molecular pattern targeted by human defensins
Source: Sci Rep. 2016 Sep 1;6:32499. doi: 10.1038/srep32499 (PMC5007486; doi:10.1038/srep32499)
Supplement: Supplementary Information [file srep32499-s1.pdf]

Supplementary Information

Thermodynamic instability of viral proteins is a pathogen-associated molecular pattern targeted by human defensins

Elena Kudryashova, Pratibha C. Koneru, Mamuka Kvaratskhelia, Adam A. Strömstedt, Wuyuan Lu, Dmitri S. Kudryashov

Supplementary Figure S1

Supplementary Figure S2

Supplementary Table S1

Supplementary Figure S1

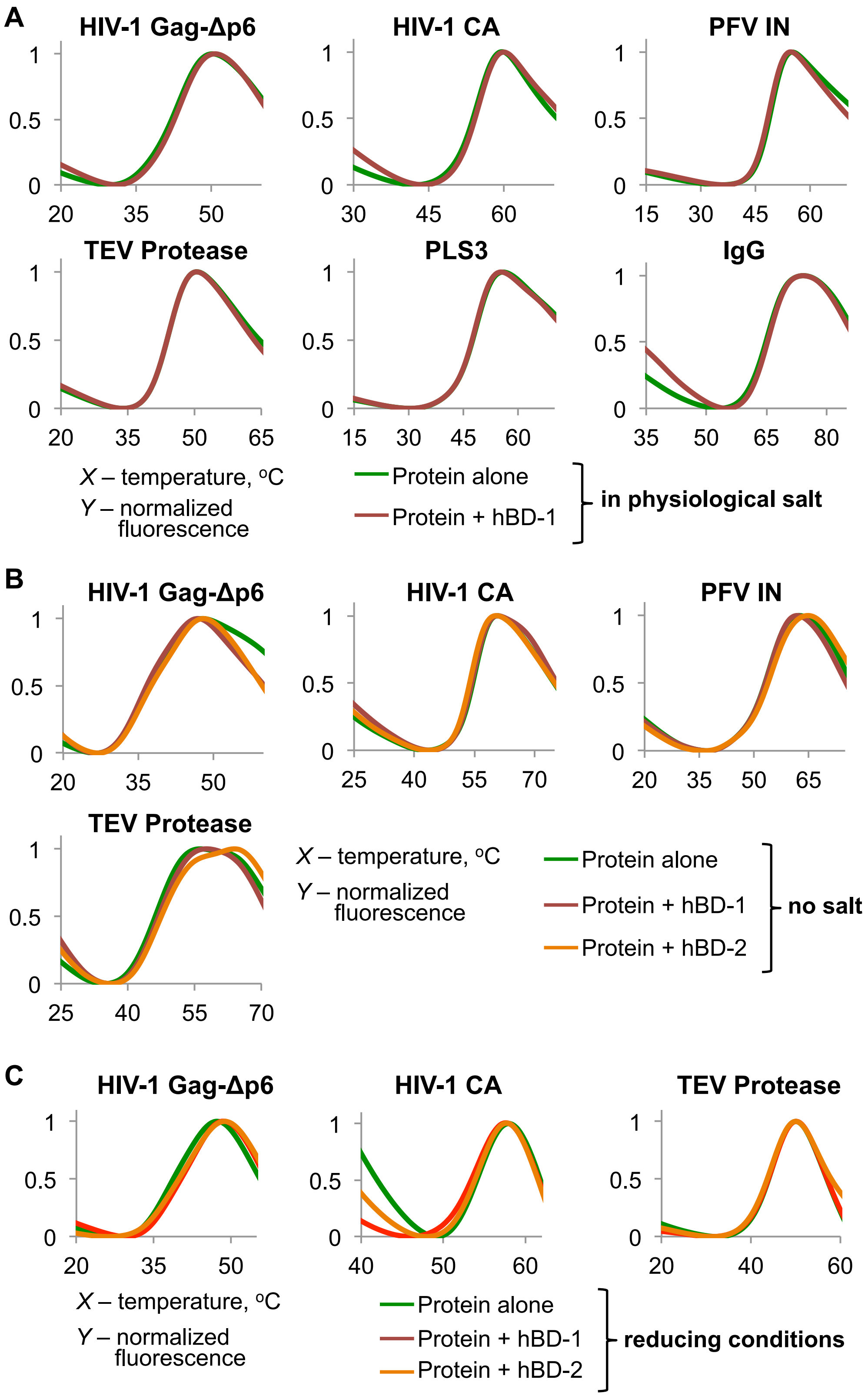

Supplementary Figure S1. β-Defensins do not cause unfolding of viral proteins. (**A**) Thermal denaturation profiles of viral proteins (HIV-1 Gag-Δp6, HIV-1 CA, PFV IN, and TEV protease) and mammalian proteins (PLS3 and IgG) were tested in the absence (green lines) and presence of 3-molar excess of β-defensin hBD-1 (red lines) at physiological salt concentration. (**B**) In the absence of salt (20 mM HEPES, pH 7.5), β-defensins hBD-1 and hBD-2 (orange lines) do not affect melting temperatures of the tested proteins (HIV-1 Gag-Δp6, HIV-1 CA, PFV IN, and TEV protease). (**C**) Under reducing conditions (10 mM TCEP), β-defensins hBD-1 and hBD-2 do not affect melting of the tested proteins (HIV-1 Gag-Δp6, HIV-1 CA, and TEV protease).

Supplementary Figure S2

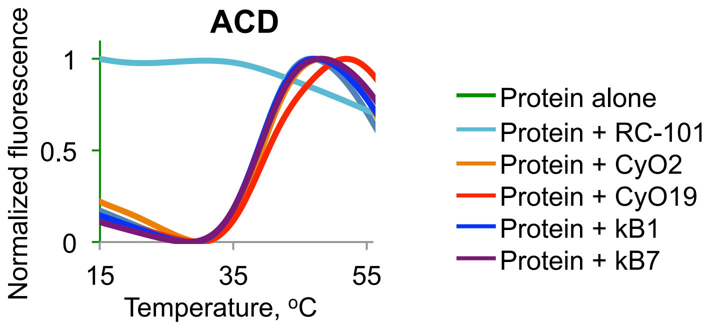

Supplementary Fig. S2. Cyclotides do not cause unfolding of a thermolabile bacterial protein toxin. DSF thermal denaturation profile of Actin Crosslinking Domain (ACD) toxin form *V. cholerae* was shifted toward lower temperature in the presence of 5-molar excess of RC-101, but not in the presence of any of the tested cyclotides (in 5-molar excess to the toxin).

Supplementary Table S1. Anti-microbial peptides (AMP) used in this study

AMP name	AMP sequence / (total number of residues)	Arg/10[1]	Lys/10[2]	His/10[3]	"+"/10[4]	"-"/10[5]	GRAVY[6]	Monomer MW, kDa[7]
HNP-1	A**C**Y**C**RIPA**C**IAGERRYGT**C**IYQ GRLWAF**CC** (30)	1.33	0	0	1.33	0.33	0.300	3.45
HD-5	AT**C**Y**C**RTGR**C**ATRESLSGV**C**EI SGRLYRL**CC**R (32)	1.88	0	0	1.88	0.63	-0.113	3.59
hBD-1	DHYN**C**VSSGGQ**C**LYSA**C**PIFTK IQGT**C**YRGKAK**CC**K (36)	0.28	1.11	0.28	1.39	0.28	-0.272	3.93
hBD-2	GIGDPVT**C**LKSGAI**C**HPVF**C**PR RYKQIGT**C**GLPGTK**CC**KKP (41)	0.49	1.21	0.24	1.71	0.24	-0.102	4.33
RC-101	GI**C**R**C**I**C**GKGI**C**R**C**I**C**GR (18; cyclic)	1.67	0.56	0	2.22	0	0.778	1.91
cyO2	GIP**C**GES**C**VWIP**C**ISSAIG**C**S**C**K SKV**C**YRN (30; cyclic)	0.33	0.67	0	1.00	0.33	0.443	3.16
cyO19	GTLP**C**GES**C**VWIP**C**ISSVVG**C**S **C**KSKV**C**YKD (31; cyclic)	0	0.97	0	0.97	0.65	0.471	3.25
kB1	GLPV**C**GET**C**VGGT**C**NTPG**C**T**C** SWPV**C**TRN (29; cyclic)	0.34	0	0	0.34	0.34	0.152	2.92
kB7	GLPV**C**GET**C**TLGT**C**YTQG**C**T**C** SWPI**C**KRN (29; cyclic)	0.34	0.34	0	0.68	0.34	0.038	3.10

Acidic residues are shown in red, basic residues are in blue, hydrophobic uncharged residues are in green, cysteines are in bold.

Parameters calculated using ExPASy ProtParam tool:

[1] – Number of arginines per 10 residues
[2] – Number of lysines per 10 residues
[3] – Number of histidines per 10 residues
[4] – Number of positively charged residues per 10 residues
[5] – Number of negatively charged residues per 10 residues
[6] – Grand average of hydropathicity (GRAVY)
[7] – Molecular weight (MW), kDa
